# Bone age recognition based on mask R-CNN using xception regression model

**DOI:** 10.3389/fphys.2023.1062034

**Published:** 2023-02-14

**Authors:** Zhi-Qiang Liu, Zi-Jian Hu, Tian-Qiong Wu, Geng-Xin Ye, Yu-Liang Tang, Zi-Hua Zeng, Zhong-Min Ouyang, Yuan-Zhe Li

**Affiliations:** ^1^ Department of Radiology, Guangzhou Twelfth People’s Hospital, Guangzhou, China; ^2^ Department of Radiology, The Fifth Affiliated Hospital of Guangzhou Medical University, Guangzhou, China; ^3^ Department of CT/MRI, The Second Affiliated Hospital of Fujian Medical University, Quanzhou, China

**Keywords:** hand bone X-ray images, bone age assessment, deep learning, mask R-CNN, Xception

## Abstract

**Background and Objective:** Bone age detection plays an important role in medical care, sports, judicial expertise and other fields. Traditional bone age identification and detection is according to manual interpretation of X-ray images of hand bone by doctors. This method is subjective and requires experience, and has certain errors. Computer-aided detection can effectually enhance the validity of medical diagnosis, especially with the fast development of machine learning and neural network, the method of bone age recognition using machine learning has gradually become the focus of research, which has the advantages of simple data pretreatment, good robustness and high recognition accuracy.

**Methods:** In this paper, the hand bone segmentation network based on Mask R-CNN was proposed to segment the hand bone area, and the segmented hand bone region was directly input into the regression network for bone age evaluation. The regression network is using an enhancd network Xception of InceptionV3. After the output of Xception, the convolutional block attention module is connected to refine the feature mapping from channel and space to obtain more effective features.

**Results:** According to the experimental results, the hand bone segmentation network model based on Mask R-CNN can segment the hand bone region and eliminate the interference of redundant background information. The average Dice coefficient on the verification set is 0.976. The mean absolute error of predicting bone age on our data set was only 4.97 months, which exceeded the accuracy of most other bone age assessment methods.

**Conclusion:** Experiments show that the accuracy of bone age assessment can be enhancd by using the Mask R-CNN-based hand bone segmentation network and the Xception bone age regression network to form a model, which can be well applied to actual clinical bone age assessment.

## 1 Introduction

Bone age can reflect the level and maturity of human growth and development. Bone age detection is widely used in clinical medicine (Wilson, 1999), forensic medicine, sports medicine and other fields. In clinical medicine, endocrine, developmental and nutritional disorders can be diagnosed by assessing skeletal development. Through bone age, we can determine the development stage of adolescents and children, determine the appropriate time for orthopaedic surgery such as teeth or nasal cavity, and provide a basis for predicting height. In forensic science, bone age can estimate the real birth time of an individual and provide legal basis for criminal identification. In addition, according to the individual development determined by bone age, we can guide the selection of athletes more scientifically. The research on children’s bone development has a history of more than 100 years since the discovery of roentgen rays. In this long research process, in order to find the part that can best represent the individual’s age, people have conducted extensive research on the ossification law of the ossification center of each joint of the human body, and put forward various bone evaluation methods according to the ossification law of different parts. Because the bone development of the wrist can represent the whole body bone, and the radiation damage to the human body is the least when taking X-rays, it has become the main part of the application of bone age evaluation. The traditional bone age recognition method - bone age standard atlas and scoring method is an evaluation method using wrist bone analysis.

By observing the shape, size and closure of different bones, the bone age can be estimated using the corresponding evaluation methods. There are many traditional bone age assessment methods, generally including counting method, Atlas method and scoring method, among which the most commonly used are GP Atlas method (Radiographic Atlas, 1959), TW2 scoring method (Paul, 1986) and TW3 scoring method (Carty, 2002). The counting method first needs to count the time when the ossification center appears and the number of bones mature. By comparing with the standard number, we can infer the bone age, but the application range is narrow and the error is large, so it has been rarely used at present. The atlas law requires comparing the X-ray film of the detector’s hand with the standard bone age atlas to infer the bone age. The advantage of Atlas method is that it pays attention to the number, shape and size of bones at the same time, but the X-ray films of some testers are quite different from the standard atlas, which is easy to be affected by subjectivity, resulting in poor accuracy; The scoring method is mainly to divide the development status of each bone in the hand into different grades, and then evaluate the corresponding grades and scores of different bones. The sum of the scores of all bones is the final score of the hand X-ray film. Finally, the bone age can be inferred according to the median curve of bone maturity score. At present, TW3 scoring method is recognized as one of the more accurate methods. These methods require high professional skills of orthopedic doctors, require doctors to analyze and calculate quantitative indicators for a long time, and are very vulnerable to subjective factors, resulting in excessive operator error. As a result, research on automated bone age assessment method has been increasing.

With the popularization and development of computer technology, the emergence of computer-aided bone age prediction technology solves the problems of great influence and randomness by doctors’ subjective factors in the traditional bone age prediction, and correspondingly reduces the time of bone age prediction. However, the early computer-aided bone age prediction method has some disadvantages, such as large error and small scope of application (Thodberg et al., 2009a). In recent years, computer vision and image recognition technology have developed rapidly. Especially with the disclosure of a large number of hand bone X-ray datasets and the remarkable improvement of computer performance, machine learning-based bone age prediction has also become a research hotspot in recent years.

In 2007, Hsieh et al. (2007) pointed out that bone age can be evaluated according to the geometric characteristics of carpal bones. The shape and area of wrist bone are extracted manually, and the bone age classification of image is realized by using linear classifier, radial basis function and principal component analysis. The feasibility of bone age recognition by segmenting wrist region is preliminarily proved. In 2010, (Chi-Wen et al., 2010) used morphological methods to extract the shape of wrist bone, and used fuzzy classifier and principal component analysis to infer bone age. Bone age is calculated by considering the geometrical features of the carpal bones. The main characteristics include the area and proportion of each bone of carpal bone, as well as the contour information of carpal bone. Thodberg et al. (2009b) developed a bone age evaluation system called bonex PERT by combining G & P method and TW2 method. It was tested on 1559 X-ray private data sets of hand bones of children aged 7 to 17, and its mean square error (MSE) was 9.6 months. Lee et al. (2017) developed a system for dividing bone age in units of age. Tested on private data sets, the MAE of women and men reached 11.16 months and 9.84 months respectively. SpampinatoPalazzoGiordano et al. (2017) used Bonet, an end-to-end convolutional neural network with an MAE of 9.48 months, in the DHA (digital handatlas) public dataset. Larson et al. (2018) proposed a new ResNet-50-based method for skeletal age estimation. The MAE on RSNA (Radiological Society of North America) children’s bone age challenge test set was 7.2 months, and the MSE (Halabi et al., 2019) on DHA subset was 8.76 months. Sung et al. (2019) used a Faster R-CNN network to detect regions of interest (ROI) in TW3 and converted 13 maturity predictions into bone age. The MAE on its internal test set was 5.52 months. Bui et al. (2019) also used Faster R-CNN network to detect ROI in TW3, and then trained a support vector regression model to predict bone age, which was tested on DHA data set, and its MAE was 7.08 months. Baoyu et al. (2019) uses the FasterR-CNN network to measure the ossification centers of the epiphysis and carpal bones, replaces the convolutional part in Bonet with ResNet-50, and conducts experiments on DHA. Its MAE was 6.12 months. Xu et al. (2020) first extracted local binarized features from the image, and then used SVM for classification. The MAE tested on the private data set was 5.46 months.

Although the above method can reduce the workload of manual work to a certain extent and achieve the purpose of bone age assessment, there are still three problems: 1) Some methods are to evaluate the X-ray image of the entire hand bone, resulting in too much background information in the image, resulting in serious errors; some inspection methods are to extract the clinical RoI of the hand bone in advance, so it is difficult to avoid manual operation; 2) For some detection methods, the first part is to preprocess bone X-rays, and the second part is to use feature information to evaluate bone age, which greatly restricts the automatic evaluation of bone age; 3) The evaluation error is high.

To solve the above problems, this paper proposes a composition model of hand bone segmentation network based on Mask R-CNN and xception bone age regression network to evaluate bone age, overcomes the limitations and complexity of traditional manual extraction techniques, improves the accuracy of bone age, and is more suitable for clinical bone age evaluation than other evaluation methods.

## 2 Methodology

### 2.1 Deep learning

The concept of deep learning was first proposed by Hinton from the University of Toronto, Canada. Deep learning emerges from human neural networks. It simulates how the human cerebral cortex and visual nervous system process information, analyzes and interprets data (Hu et al., 2017).

Deep learning is an algorithm that uses complex structures or multiple non-linear neural networks to represent and learn the provided data by setting perceptrons with different numbers of hidden layers, so as to automatically obtain the appropriate link weights, and apply them to classify and identify the original data, so that the computer can simulate the human brain to realize hierarchical processing and understanding of data. The “depth” of deep learning is relative to the “shallow” methods such as support vector machine, lifting method and maximum entropy method (Hatcher and Yu, 2018). The shallow learning method mainly extracts the sample features manually, which can only obtain the image representation features. The deep neural network obtains the features of the original data layer by layer through multiple non-linear network structure, and obtains the hierarchical feature expression in the way of automatic learning.

**FIGURE 1:**
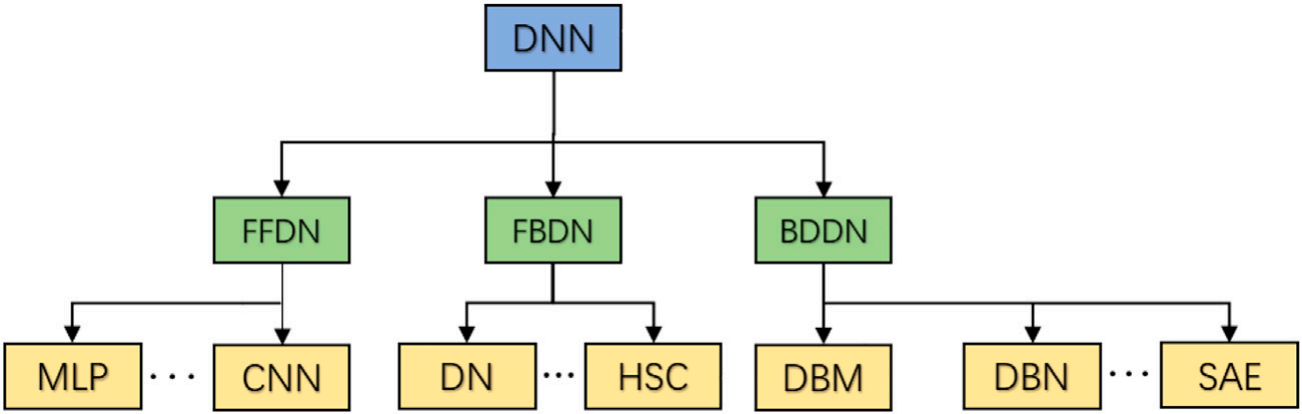
Deep neural network classification structure.

Deep learning has shown its unique advantages in search technology, data mining, machine learning, machine translation, natural language processing, multimedia learning and so on. According to the different characteristics of depth learning algorithm, it is divided into feedforward depth network (FFDN), feedback depth network (FBDN) and bidirectional depth network (BDDN). According to the network structure division, the current main deep neural networks are roughly classified as shown in Figure 1. Feedforward depth network refers to the one-way data input through one or more hidden layer perceptrons to the output layer, including multi-layer perceptron (MLP) and convolutional neural network (CNN) (Hu et al., 2017). CNN network came out earlier and has been widely used in computer vision tasks.

### 2.2 Convolutional neural network

Convolutional neural networks (CNN) is a kind of feedforward neural networks with depth structure and convolution calculation. It is one of the representative algorithms of deep learning.

A convolutional neural network usually consists of input layer, hidden layer and output layer. The input layer of a convolutional neural network can handle multidimensional data. The structure and working principle of the output layer are similar to the traditional feedforward neural network. In an image classification problem, the output can be a classification label. In the object recognition problem, the output can be the center coordinates, size and classification of the output object. . Hidden layers usually include convolutional layers, pooling layers and fully connected layers, which are introduced as follows:

The main function of the convolutional layer is to extract the input data. The number of convolution kernels determines the number of convolution output channels in advance. On the feature map output by the previous convolutional layer, translate and slide according to the set step size, multiply and sum the position elements corresponding to the convolution kernel, and finally output the feature map of the next layer. The activation function layer in the convolution layer can non-linear map the convolution operation results, and map the convolution or pooled output results to a specific range, generally between 0 and 1. Therefore, it is often used in combination with convolution operation. The most frequently used activation functions include sigmoid function, hyperbolic tangent function, corrector linear unit (relu), leaky relu (lrelu), etc.

The pooling layer is also called the lower sampling layer. The most important thing is the pooling function. The result of a single point in the characteristic graph can be replaced by the characteristic graph statistics of its adjacent regions. Because the feature map after convolution layer obtains a large number of features with local correlation, if these features are directly used for training, it will cause the phenomenon of over fitting. Through the pooling layer, the large-size feature map can be represented by the small-size feature map, so as to remove the unnecessary redundant features for identifying objects, reduce the feature dimension and simplify the network calculation complexity.

The full connection layer connects each network node with each node of the previous field. Thus, the features extracted from the front can be integrated to form a feature vector. According to the difference between feature vectors and the comparison of distance, the final result can be classification operation or regression task.

### 2.3 Traditional methods of bone age assessment

The traditional assessment methods of bone age mainly use two methods: Greulich & Pyle (1959) (G&P) and Tanner-Whitehouse (TW2). Doctors usually use the first GP method to diagnose bone age. The GP method is also called the G-P atlas method. The target X-ray film was compared with the reference map to draw the conclusion. Although it is convenient, it has obvious shortcomings. It is very subjective and has high requirements for reference maps. Reference maps of different countries and races cannot be used universally (Berst et al., 2001). Tanner J and Whitehouse R developed the TW2 method to reduce the influence of human subjective factors in the evaluation of bone age. The TW2 method, also known as the Bone Age Score, differs from the previous overall comparison by comparing bones by bone. According to the development degree of each skeleton and its corresponding score, the final conclusion is calculated, and it has a higher accuracy than the G-P atlas method (King et al., 1994). Although the TW method is more objective than the G-P method, it also takes longer to evaluate once, and it does not completely escape the subjective perception of humans (comparison by skeleton still requires human effort). Helen (2002) enhancd the TW2 method and proposed the TW3 method, which made it take a little less time. In the 21st century, Heyworth B E has further shortened the time required for an evaluation using these two methods (Heyworth et al., 2013), since its main improvement is to define some shorthand methods for specific hand bone evaluation, so it still does not get rid of the human hand intervention.

In the study of automatic bone age evaluation methods, BoneXpert is the first influential automatic bone age assessment method for children in recent years, and it also has a considerable high accuracy rate. BoneXpert requires an active appearance model (AAM) that can be used to quantify shape. The earliest AAM was used for statistical modeling of human faces (Cootes et al., 2001). In BoneXpert, AAM was used to find and extract the 15 required bones in the hand bones and then score them (G-P atlas or TW scoring) using shape, strength and texture features. Compared with the traditional bone age evaluation method, this method is free from the constraints of manpower and has high accuracy. But its judgment needs to rely on some connection between real age and bone age, and because of this, it does not belong to direct judgment. Since then, many other methods of automatic bone age evaluation have emerged. For example, the carpal bones are extracted by edge detection, a model function is established, and the bone age is evaluated according to the characteristics of the carpal bones. As children grow older, the changes in carpal bone characteristics are not obvious, so this method is only useful in the evaluation of bone age in children 7 years old and younger (Zhang and GertychLiu, 2007; Somkantha et al., 2011). Other methods include fully connected neural networks using fixed-size feature vectors for description screening by means of SVD (Seok et al., 2012), and automatic carpal region extraction using support vector regression (KashifDesernoHaak and Jonas, 2016) or random forest regression (Adeshina et al., 2014). Make predictions. These methods take BAA to a new level, which is the full development of automated bone age assessment methods. However, their development is limited by data. Without enough data, they cannot have good training results, and the validation is also lacking in robustness and convincing.

### 2.4 Hand bone segmentation based on mask R-CNN

In recent years, due to the rapid development of big data, machine learning and other technologies, it has also been applied in bone age evaluation. Most hand bone X-ray images contain redundant background information (identification, artifact, noise, etc.), resulting in the regression network paying too much attention to other parts of the X-ray image except the hand bone, which affects the bone age evaluation results. It is necessary to segment the complete hand bone area to eliminate these interferences. Mask R-CNN is one of the most advanced image segmentation algorithms, so this paper uses Mask R-CNN to segment the complete hand bone region.

**FIGURE 2:**
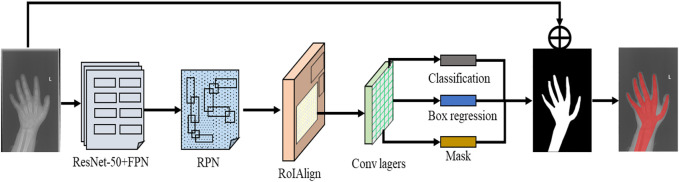
Structure of hand bone segmentation network.

Mask R-CNN has added a branch of prediction segmentation mask based on the multi category classification and candidate box regression realized by Faster R-CNN (Ren et al., 2017). Faster R-CNN first extracts the feature map of the image through ResNet-50 and feature pyramid networks (FPN), and then generates the detection frame through regional proposal networks (RPN). Faster R-CNN uses roipooling to realize the mapping of ROI from the original image area to the convolution area, pool it into a fixed size, and normalize the size of the input area into the input size of the convolution network. In the process of normalization, it is difficult to avoid the problem that the extracted features do not coincide with ROI, resulting in the loss of features. In order to solve this problem, Mask R-CNN proposes the concept of roialign, which uses the roialign layer to correct the deviation between the extracted features and the input ROI, that is, bilinear interpolation is used to calculate the eigenvalues obtained from four fixed sampling points in the ROI, and the results are fused to obtain the position of the center point. In addition, the branch used to predict the segmentation mask is essentially a full convolutional networks (FCN). FCN solves the problem of image segmentation at the semantic level by classifying the image at the pixel level. The mask obtained from the X-ray image of hand bone segmented by Mask R-CNN is fused with the original image to obtain a complete hand bone area without redundant information. Its structure is shown in Figure 2.

#### 2.4.1 Mask R-CNN loss function

Neural network training is a process of using back propagation algorithm to optimize the parameters in the network structure to reduce the loss. Loss is the penalty caused by inaccurate prediction in the process of neural network training, which describes the gap between the network prediction results and the actual results. The loss in the training process is calculated by the loss function. For each region of interest, the total loss of Mask R-CNN is defined as

$$L=L_{c}+L_{1}+L_{m}$$ (1)

Where L is the total loss of the network; $L_{c}$ refers to classification loss, which is used to measure the accuracy of network classification; $L_{1}$ is the regression loss, which is used to measure the accuracy of frame positioning; $L_{m}$ is the mask loss, which is used to measure the accuracy of mask position.

For each detection category u , calculate $L_{c}$ through the logarithm of softmax loss function

$$L_{c}\left(p,u\right)=-\log_{2}\left(p_{u}\right)$$ (2)

Calculate $L_{1}$ by ${smooth}_{L_{1}}$ loss function

$$L_{1}\left(t^{u},v\right)=\underset{t\in \left\{x,y,w,h\right\}}{\sum }{smooth}_{L_{1}}\left(t_{t}^{u},v_{i}\right)$$ (3)

In the formula: $p=\left(p_{0},\ldots ,p_{k}\right)$ is the calculation result of the SoftMax function; $v=\left(v_{x},v_{y},v_{w},v_{h}\right)$ is the real bounding box coordinates of the target to be tested; $t^{u}=\left(t_{x}^{u},t_{y}^{u},t_{w}^{u},t_{h}^{u}\right)$ , which is the coordinate correction of the bounding box for the u -th target.

The ${smooth}_{L_{1}}$ loss function is defined as

$${smooth}_{L_{1}}\left(x\right)=\left\{\begin{array}{l} 0.5x^{2}\;\;\left|x\right|<1\\ \left|x\right|<0.5\;Otherwise\; \end{array}\right.$$ (4)

Similar to $L_{c1}$ , $L_{m}$ is calculated by averaging binary cross-entropy loss function.

### 2.5 Xception regression network

Xception network is a kind of network for image classification, which is enhancd based on inception V3 (Tao and Mughees, 2021). Xception network replaces the convolution in the original inception V3 with deep separable convolution, which increases the network width and reduces the amount of parameters and calculations of the model (Chen et al., 2020). By introducing a residual connection mechanism similar to ResNet, the convergence of the network is improved, the classification accuracy is improved, and the detailed characteristics of the network are improved. In this way, the Xception network can effectively improve the performance of the model without increasing the network complexity.

**FIGURE 3:**
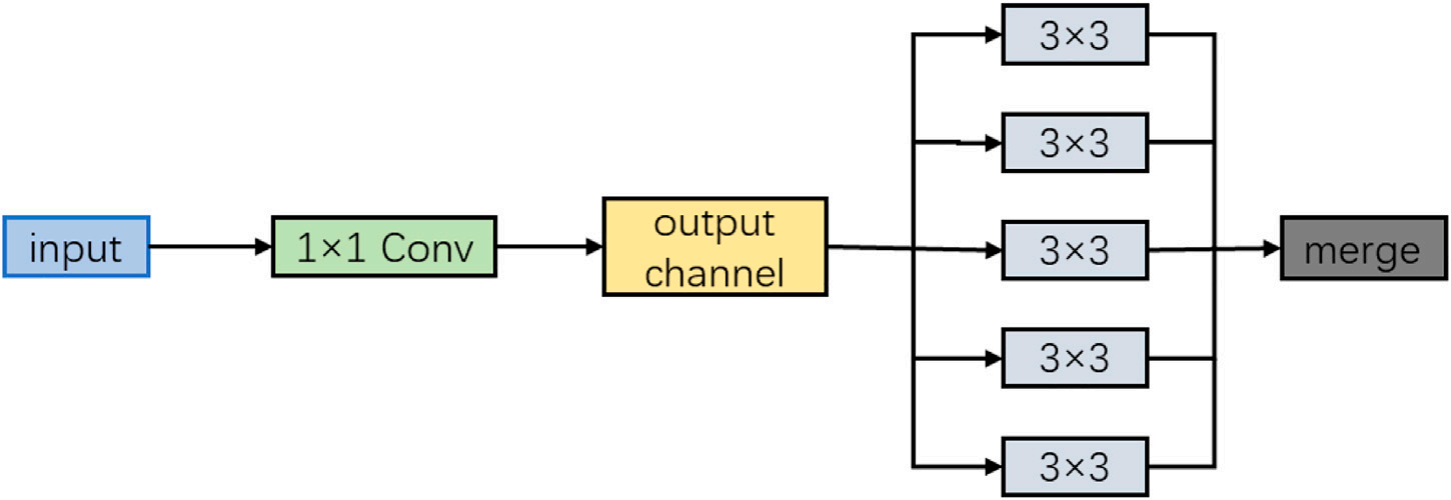
Schematic diagram of Xception.

Inception V3 divides 3×3 convolutions into 3 groups. If the feature maps of $k_{1}$ channels obtained from 1×1 of Inception V3 are completely separated, that is, $k_{1}$ different convolutions are used to convolve on m channels respectively, Then its number of parameters is $m\times k_{1}+k_{1}\times 3\times 3$ . If the number of convolution channels of InceptionV3 is set to k2, that is, the number of parameters is $m\times k_{2}+k_{2}\times 3\times 3$ . The number of parameters is $1/k_{1}$ of ordinary convolution, and this form is called Extreme Inception, or Xception for short, as shown in Figure 3:

Xception model is divided into three parts: entry flow, middle flow and exit flow. There are 14 blocks in total, including 4 input flows, 8 intermediate flows and 2 output flows (Chollet, 2017).

The improvement of Xception is to introduce deep separable convolution and residual connection. Deep separable convolution can reduce the amount of model parameters and maintain high accuracy. Residual connections can solve the vanishing or exploding gradient problem.

## 3 Results

### 3.1 Evaluating indicator

The hand bone segmentation network uses the Dice coefficient as the evaluation index of the hand bone region segmentation result. The Dice coefficient is mainly used to calculate the coincidence degree of the prediction mask and the label mask. The operation process is shown in Eq. 5:

$$Dice=\frac{2\left|A\cap B\right|}{\left|A\right|+\left|B\right|}$$ (5)

Among them, A stands for the pixel area of the label mask, and B stands for the pixel area of the prediction mask. The larger the value of the Dice coefficient, the better the segmentation effect.

The bone age regression network uses MAE as the evaluation index of the bone age evaluation results, and the operation process is shown in Eq. 6:

$$MAE=\frac{1}{N}\overset{N}{\underset{i=1}{\sum }}\left|{\hat{y}}_{i}-y_{i}\right|$$ (6)

Among them, N indicates the total number of samples, $y_{i}$ indicates the true value of bone age, and ${\hat{y}}_{i}$ indicates the predicted value of bone age. The lower the MAE value, the better the prediction effect can be obtained.

### 3.2 Data set

**FIGURE 4:**
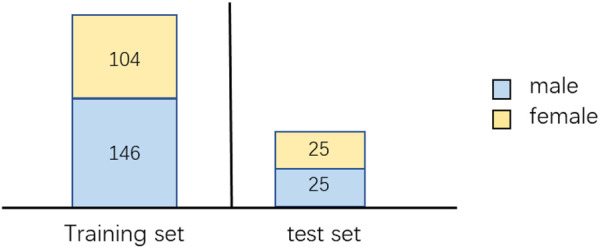
Number of samples for male and female in training and test sets.

**FIGURE 5:**
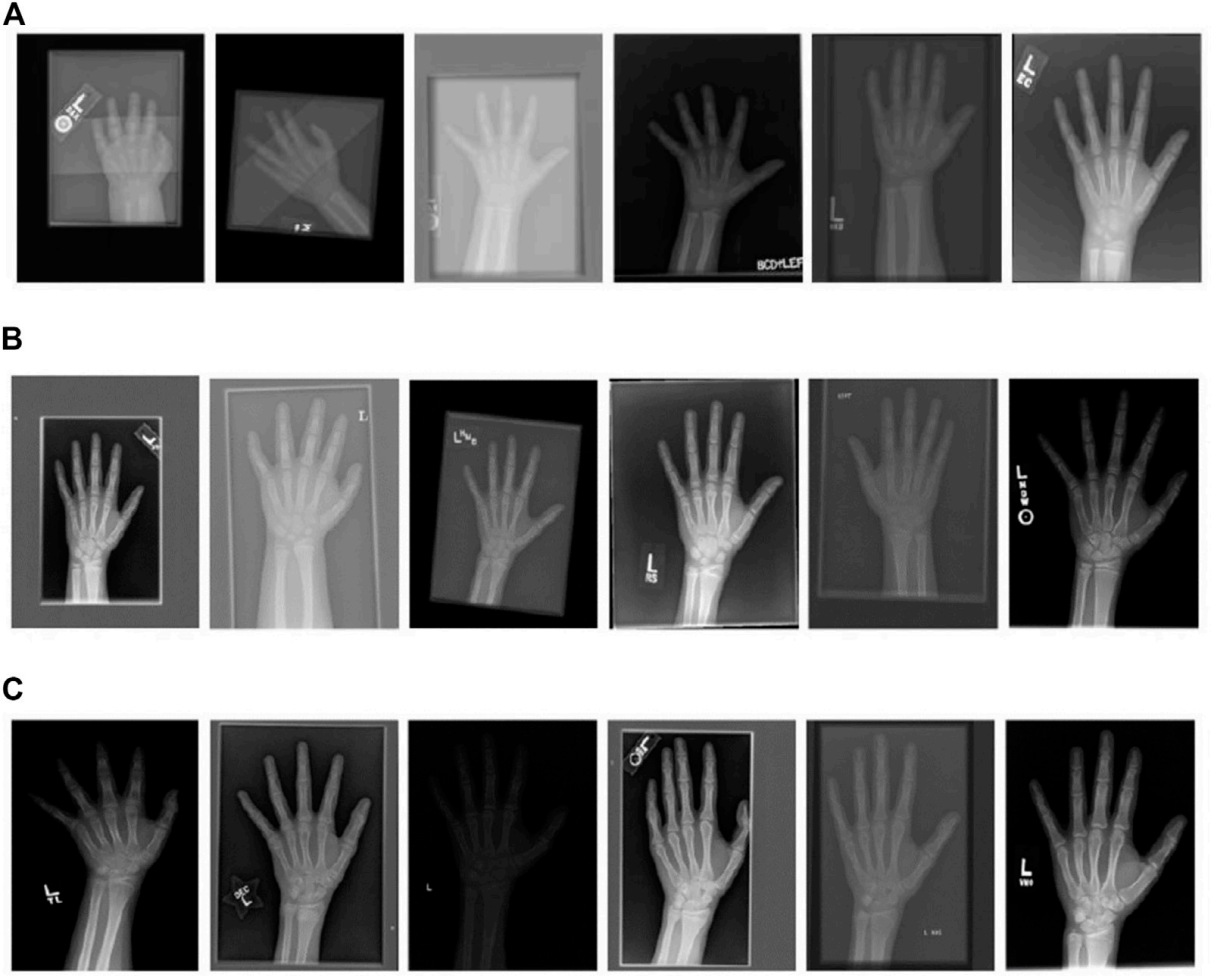
X-rays of hands based on **(A)** 1–6 years old; **(B)** 7–12 years old; **(C)** 13–18 years old.

X-ray films of the hand bones of human subjects were scanned and consolidated by the Department of Radiology, Guangzhou Twelfth People’s Hospital, annotated by multiple experts, and finally the average value of the predicted age of multiple experts was taken as the label of the data. The data set includes 300 children’s hand X-ray films, of which the training set contains 250 images, including 146 for men and 104 for women, 50 for test set, 25 for men and 25 for women respectively. The data distribution is shown in Figure 4 In the training set and test set, the data distribution is similar. The age of most samples is about 6–15 years old, and there are few data before 0–6 years old and after 18 years old. The age distribution of male and female data is shown in Figure 5.

The image quality of the hand bone data sets varies greatly, and the image resolution is inconsistent. The maximum resolution of the image in the training set reaches 2,970 × 2,460, while the smallest resolution is only 1,011 × 800, palm posture is also different, and there are problems such as rotation, turnover, occlusion and inconsistent size. Through the observation of X-ray films of hand bones at different ages, it can be clearly seen that hand bones show different shape and size characteristics at different ages. In the stage of 1–6 years old, the gap between metacarpal bone and phalange is large, calcification points and ossification centers in the wrist have just appeared, a small number of bones such as cephaloid bone and uncinate bone are scattered in the wrist area, and ossification centers begin to appear in the radius. At the age of 7–12 years, the ossification center increases gradually, all carpal bones are basically mature, and the ulnar epiphysis has just begun to develop. At the age of 13–18, almost all bones begin to heal, so the gap between joints becomes smaller and the morphological differences between bones are smaller.

### 3.3 Preprocessing and data augmentation

Data preprocessing plays an important role in the method based on deep learning, because the quality of data directly determines the upper limit of the final result. The data preprocessing method in this paper is histogram equalization. Histogram equalization can enhanc the brightness of dark image and reduce the impact of data quality on the model. Histogram equalization needs to first calculate the pixel histogram of the image, then make it evenly distributed in the appropriate interval through the gray conversion function, and finally process the original image based on the adjusted histogram to make the image clearer.

For an image with a gray level of $\left[\begin{array}{cc} 0, & L-1 \end{array}\right]$ , the frequency of occurrence of pixels needs to be counted first:

$$P\left(r_{k}\right)=\frac{n_{k}}{MN},\;k=0,1,2,\ldots ,L-1$$ (7)

In the Equation: M and N stand for the length and width of the image respectively, $r_{k}$ stands for the *k*th grayscale value, and $n_{k}$ stands for the number of occurrences of the pixel with the grayscale value $r_{k}$ .

Then we can use Eq. 8 to calculate the original gray level corresponding to the equalized gray level:

$$s_{k}=T\left(r_{k}\right)=\left(L-1\right)\overset{k}{\underset{j=0}{\sum }}P\left(r_{j}\right)=\frac{L-1}{MN}\overset{k}{\underset{j=0}{\sum }}n_{j},\;k=0,1,2,\ldots ,L-1$$ (8)

After histogram equalization, the image can more clearly display the structure and texture of carpal bone, and the distribution of pixels is more uniform.

In the process, the model is prone to over fitting, which makes the trained model unable to be generalized to new data. Therefore, data enhancement technology needs to be used to increase samples. In this experiment, the input images of each batch are randomly turned horizontally and rotated by 20°, and the training samples and verification samples are expanded to improve the model’s generalization ability to new data.

### 3.4 Mask R-CNN segmentation results

**FIGURE 6:**
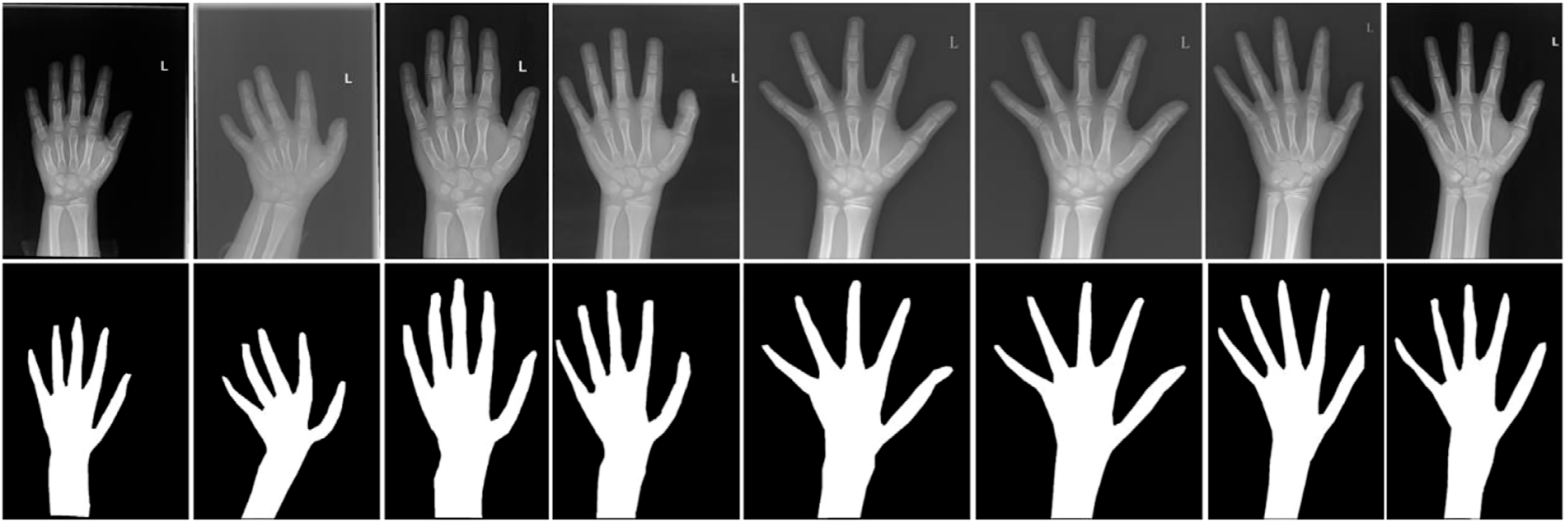
Results of hand bone segmentation.

The average Dice coefficient of the hand bone segmentation network model on the validation set is 0.976, and then the optimal model is used for the hand bone segmentation of the remaining 250 hand bone X-ray images. Part of the segmentation results are shown in Figure 6. The hand bone area can be segmented to eliminate the interference of redundant background information.

### 3.5 Evaluation results based on xception regression model

The performance of our method is verified by ablation experiments. In Model 1, bone age is directly measured using the original Xception network, resulting in a MAE of 8.18 months. In Model 2, Mask R-CNN is used to first segment the hand bone area, and then Xception neural network is used to estimate the bone age, and the final MAE is 6.03 months. Model 3 first uses Mask R-CNN to segment the hand bone area, and then uses the modified Xception network to measure the bone age, and the result is 5.44 months. The results of bone age determination in the ablation test are shown in Table 1.

**TABLE 1:** Comparison of bone age assessment results in ablation experiments (√ means using the model, × means not using the model).

Model	Xception	Mask R-CNN	Improved	MAE/months
1	√	×	×	8.18
2	√	√	×	6.03
3	√	√	√	5.44

## 4 Discussion

Bone age is the age description of bone development process, which stands for the general state of bone development of normal people of different ages. Bone age has a position that cannot be underestimated in medical and healthcare, sports, and judicial appraisal. A method commonly used internationally to assess the physical development level of adolescents is to use X-ray images of the hands to assess bone age.

Computer-aided detection can effectively enhanc the efficiency of medical diagnosis, quickly screen and discriminate in huge medical data, and can reduce the problem of low efficiency of manual identification and different results for different readers. At the same time, in addition to the field of sports, in view of the problem of low detection accuracy caused by the difficulty of data acquisition and inconsistent standards in small hospitals, judicial detection and other fields, this paper starts from the preprocessing of medical images and the improvement of deep detection model algorithms. Using deep learning, the composition model of the hand bone segmentation network and Xception bone age regression network based on Mask R-CNN is proposed. The experiments show that the method in this paper is simpler and more accurate than other methods, and has achieved satisfactory results, but there are still many places to be. Further improvement, follow-up work can be carried out from the following aspects.

1) Metacarpal, phalangeal, ulnar or radial bones are used for bone age recognition. This paper mainly studies the bone age recognition of hand data. The effect of bone age recognition of other local bones needs further research and experiment.
2) Solve the problem of uneven number of data sets at different ages. The quality of data set directly affects the results of bone age prediction model, and the insufficient amount of data will also cause many problems. Due to the limitation of the actual scene, the number of X-rays taken by children and adults is small, resulting in a small number of low age and high age stages. Therefore, there are problems of over fitting the data of the middle age group and under fitting the data of the low age group and high age group. The accuracy of bone age recognition can be further improved by designing appropriate data sampling strategies and data enhancement methods.
3) Model pruning. By designing lightweight networks or training complex networks and then pruning, the time of model testing can be reduced and the efficiency of bone age recognition can be further improved.
4) If conditions permit, we can try to deploy the algorithm to the actual relevant equipment, or package the model as computer software and put it into clinical application.

In summary, although the research of the paper has achieved the intended purpose, it still needs to be further improved. At present, the application of deep learning technology in bone age evaluation is still in its infancy, but its development potential is still great, requiring the joint efforts of computer scientists, medical industry experts and medical institution experts. Volumetric modelling of the hand structures (Zhao et al., 2022) assists in the analysis of the orthopedics condition. In addition to the Mask R-CNN deep learning technique, the consideration of implementing extreme learning (Lu et al., 2020; Tang et al., 2022) for the image classification is proposed as future work, and will enhance the current medical diagnosis, learning the different possible clinical treatment (Shen et al., 2015), as well as provide improvement in structural mechanics (Wong et al., 2012) of the bone or related tissues for research applications.

## 5 Conclusion

This paper proposes an automatic bone age assessment method based on deep convolutional neural networks. Firstly, the segmentation network based on Mask R-CNN is used to accurately remove the unnecessary information in the original image and segment the complete hand bone area, which provides a good data enhancement effect for the bone age assessment network. The performance of the bone age prediction model was enhancd, and the MAE was reduced by 2.15 months compared with the image noise without image noise removal. Then, the improved Xception regression network model was introduced, and the final MAE was 5.44 months, which has a relative reduction of 2.74 months. The experimental results show that, compared with other methods, this method is simpler and more accurate, can significantly improve the recognition rate of bone age, and has good application value in clinical application.

## Data Availability

The raw data supporting the conclusion of this article will be made available by the authors, without undue reservation.
